# The Line between Psychosis and Schizotypy: a case report

**DOI:** 10.1192/j.eurpsy.2022.1110

**Published:** 2022-09-01

**Authors:** L. Huerga García, E. Hernández Padrón, N. Casanova Gracia, N. Torres Nieves, P. Gómez Pérez, F. Garcia Gómez-Pamo, J.J. Dorta Gonzalez, J.F. Dorta González

**Affiliations:** Hospital Universitario Nuestra Señora de La Candelaria, Psiquiatría, Santa Cruz de Tenerife, Spain

**Keywords:** schizotypydisorder

## Abstract

**Introduction:**

Since Kraepelin and Bleuler, schizotypy was understood as a mild expression of psychosis, a latent form with the same trajectory but different severity. They pointed characteristics such as being eccentric, unreasonable, supersticious or hipersensitive, interpersonal aversiveness (often related to suspiciousness and expectation of rejection), ambivalence, anhedonia,… and psychosis-like features that don’t usually lead to help-seeking.

**Objectives:**

To do a case review

**Methods:**

We report a case of a 17 years old boy with a childhood trauma history who started psychiatric consultations a year and a half ago because his “usual” (as his mother referred) strange behaviour got worse, which was perceived by his

ENT specialist. During the appointments, the patient showed suspiciousness, odd speech, inappropriate affect, tendency to social withdrawal, obsessive ruminations with sexual content and occasional perceptual experiences (such as depersonalization, derealization and auditory hallucinations).

**Results:**

Psychosis and schizotypy are linked historically and phenomenologically, which is evidenced by their placement in non-affective psychosis in the ICD-10 and DSM-5, and it is known that the direct observation (by clinicians or family members) during the childhood and adolescence are key for a correct diagnosis. In fact, this construct reflects a phenotypic expression of vulnerability to schizophrenia, and during childhood or adolescence it may be understood as an early mental risk state.

**Conclusions:**

In contrast to models of psychosis that mainly rely on positive features and assume a progression of them, the positive traits of schizotypy seem to be beneficial and related to a “benign or happy schizotypy” according to the articles we reviewed.

**Disclosure:**

No significant relationships.

